# Mortality after cardiac arrest in children less than 2 years: relevant factors

**DOI:** 10.1038/s41390-023-02764-2

**Published:** 2023-08-04

**Authors:** Goeun Bae, So Hyun Eun, Seo Hee Yoon, Heoung Jin Kim, Hye Rim Kim, Moon Kyu Kim, Ha Neul Lee, Hyun Soo Chung, Chungmo Koo

**Affiliations:** 1 Department of Emergency Medicine, Gabeuljangyu hospital, Gimhae, South Korea; 2 Department of Pediatrics, Severance Children’s Hospital, Yonsei University College of Medicine, Seoul, South Korea; 3 Department of Biomedical Systems Informatics, Yonsei University College of Medicine, Seoul, South Korea; 4 Department of Pediatrics, Yongin Severance Hospital, Yongin, South Korea; 5 Department of Emergency Medicine, Severance Hospital, Yonsei University College of Medicine, Seoul, South Korea; 6 Department of Pediatrics, Dankook University Hospital, Cheonan, South Korea

## Abstract

**Background:**

There are only scant studies of predicting outcomes of pediatric resuscitation due to lack of population-based data. This study aimed to determine variable factors that may impact the survival of resuscitated children aged under 24 months.

**Methods:**

This is a retrospective study of 66 children under 24 months. Cardiopulmonary resuscitation (CPR) with pediatric advanced life support guideline was performed uniformly for all children. Linear regression analysis with variable factors was conducted to determine impacts on mortality.

**Result:**

Factors with statistically significant increases in mortality were the number of administered epinephrine (*p* value < 0.001), total CPR duration (*p* value < 0.001), in-hospital CPR duration of out-hospital cardiac arrest (*p* value < 0.001), and changes in cardiac rhythm (*p* value < 0.040). However, there is no statistically significant association between patient outcomes and remaining factors such as age, sex, underlying disease, etiology, time between last normal to CPR, initial CPR location, initial cardiac rhythm, venous access time, or inotropic usage.

**Conclusion:**

More than 10 times of epinephrine administration and CPR duration longer than 30 minutes were associated with a higher mortality rate, while each epinephrine administration and prolonged CPR time increased mortality.

**Impact statement:**

- This study analyzed various factors influencing mortality after cardiac arrest in patients under 24 months.
- Increased number of administered epinephrine and prolonged cardiopulmonary resuscitation duration do not increase survival rate in patients under 24 months.
- In patients with electrocardiogram rhythm changes during CPR, mortality increased when the rhythm changed into asystole in comparison to no changes occurring in the rhythm.

## Introduction

Pediatric cardiac arrest is a significant public health issue in the United States, with over 20,000 cases reported.^1,2^ Resuscitation outcomes depend on various factors, not just patients' pre-existing conditions. In the case of adults, several studies have been conducted, such as prediction of neurological outcomes after out-of-hospital cardiac arrest and in-hospital cardiac arrest.^3,4^ Although there have been several studies of cardiac arrest in pediatric populations, limited research exists on pediatric resuscitation cases, particularly for those under 24 months of age, due to a lack of population-based data.^1–7^

Furthermore, studies based on the revised American Heart Association (AHA) guidelines in 2020 are scarce. Therefore, this study examined various factors, including etiological differences, which could impact the survival of resuscitated children under 24 months old.

## Methods

This is a retrospective analysis of data collected from Severance Children’s’ Hospital, Department of Pediatrics, from January 2010 to December 2020. This study was approved by the Institutional Review Board (4-2022-0024). Participants of this study were of the age ranging from 0 to 23 months including infants and toddlers, who had experienced cardiac arrest out-of-hospital or in-hospital at least once.

High-quality cardiopulmonary resuscitation (CPR) according to pediatric advanced life support (PALS) guidelines was performed by board-certified physicians. Misreported cases such as syncope, not cardiac arrest and cases of poor-quality CPR not following PALS guidelines were excluded. Only the initial cardiac arrest counted as a meaningful event in patients with recurrent episodes of cardiac arrest in the same admission period.

**Fig. 1:**
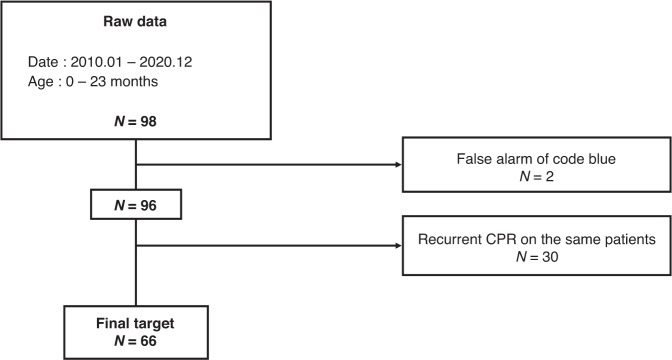
Chart depicting final participants. The flow chart depicts candidate elimination, with participants at each step were represented as "N".

In total, 98 patients were primarily selected. Two cases of misreported cases and 30 cases of recurrent CPR on the same patients were excluded. Finally, 66 children were recruited for our study, whose medical data were reviewed (Fig. 1).

Age, sex, underlying disease, etiology, time between last normal to CPR, initial CPR location, administered epinephrine, in-hospital CPR duration out-hospital cardiac arrest (OHCA), presence of doctor on duty, initial cardiac rhythm, changes and kinds of cardiac rhythm, venous access securing time, and use of inotropes were considered as the variable factors similar to the previous scoring tools used in adults^3^ to discover prognostic predictors. A good outcome is defined as the recovery of spontaneous circulation (ROSC), while a bad outcome is defined as failure of recovery including death. Outcome after ROSC was not included in the analysis.

We chose a cut-off value of 30 minutes for CPR duration and 10 as the threshold number for administered epinephrine, similar to previous studies having clinically significant outcomes.^8,9^ Epinephrine was administered at an interval of 3 minutes. The administration of epinephrine was analyzed in two ways: first, by the number of each administration as a continuous variable. Second, it was divided into two categories (less than ten times administration and more than ten times administration) and interpreted as a categorical variable.

Likewise, CPR duration was also considered as both continuous and categorical variables and divided into groups of 5 minutes each. The final changes in rhythm were analyzed by eliminating a small number of duplicates that had appeared.

### Analyses and statistical methods

Our study used the analyzing program, SAS (version 9.4, SAS Inc., Cary, NC) to statistically analyze the impact of variables and distinguish each variable according to the confirmed survival status as an outcome of cardiac arrest. The T- test was used for continuous variables while Mann–Whitney U tests for non-normally distributed factors. Chi-square test or Fisher’s exact test was used for categorical variables. Linear regression was performed to analyze the relationship between the outcome and each factor. For some factors, as the number of patients belonging to a specific category was 0 or too small, the model was obtained using the Firth method. We considered *p* value < 0.05 as statistically significant and the numbers were rounded to the second decimal place.

## Results

The characteristics of the 66 patients are described in Table 1. In the demographic data, numbers of epinephrine administered, total CPR duration, in-hospital CPR duration OHCA, and changes in cardiac rhythm showed statistically meaningful outcomes.

**Table 1:** Characteristics of patients.

Variables	Alive (*n* = 38) Mean ± SD or *n* (%)	Death (*n* = 28) Mean ± SD or *n* (%)	*p* value
Median Age (month-old)	7.6 (IQR = 4.7–13.1)	5.3 (IQR = 2.77–10.28)	0.094
Sex			
Male	23 (60.53)	15 (53.57)	0.572
Female	15 (39.47)	13 (46.43)	
Underlying disease			0.039
None	6 (15.79)	5 (17.86)	
Congenital	11 (28.95)	14 (50)	
Acquired	0 (0)	2 (7.14)	
Combined	21 (55.26)	7 (25)	
Etiology			0.326
Cardiac origin	2 (5.26)	4 (14.29)	
Pulmonary origin	21 (55.26)	12 (42.86)	
Trauma	1 (2.63)	0 (0)	
Hypovolemic	1 (2.63)	0 (0)	
Sudden infant death syndrome	5 (13.16)	7 (25)	
Multiple organ failure	5 (13.16)	5 (17.86)	
Unknown	3 (7.89)	0 (0)	
Last normal to CPR (min)	5.6 ± 29.1	3.1 ± 8.1	0.625
Initiate CPR site			0.548
OHCA	8 (21.05)	7 (25)	
Emergency department	4 (10.53)	6 (21.43)	
General ward	7 (18.42)	6 (21.43)	
Intensive care unit	15 (39.47)	9 (32.14)	
Operation room	3 (7.89)	0 (0)	
CT or MR room	1 (2.63)	0 (0)	
Median value of number of epinephrine (time)	2 (IQR = 1–5)	16.5 (IQR = 7–22)	<.001
Categorized number of epinephrine			<.001
0 ~ 10 times	32 (84.21)	10 (35.71)	
More than 10 times	6 (15.79)	18 (64.29)	
Total CPR duration (min)	16.5 ± 15.8	57.8 ± 36.6	<.001
Categorized total CPR duration			0.001
0 ~ 5 min	11 (28.95)	2 (7.14)	
6 ~ 10 min	9 (23.68)	0 (0)	
11 ~ 15 min	4 (10.53)	2 (7.14)	
16 ~ 20 min	1 (2.63)	1 (3.57)	
21 ~ 25 min	2 (5.26)	1 (3.57)	
26 ~ 30 min	3 (7.89)	3 (10.71)	
More than 31 min	8 (21.05)	19 (67.86)	
In hospital CPR duration of OHCA (min)	13.6 ± 14.2	53.5 ± 37.6	<.001
Presence of doctor on duty			0.259
Yes	16 (42.11)	8 (28.57)	
No	22 (57.89)	20 (71.43)	
Initial rhythm			0.827
Asystole	15 (39.47)	13 (46.43)	
VT	0 (0)	0 (0)	
Vf	0 (0)	0 (0)	
PEA	16 (42.11)	11 (39.29)	
Bradycardia	7 (18.42)	4 (14.29)	
Changes of rhythm			0.040
None	29 (76.30)	16 (53.30)	
To Asystole	2 (5.30)	9 (30.00)	
To VT	0 (0)	0 (0)	
To Vf	2 (5.30)	2 (6.70)	
To PEA	5 (13.20)	3 (10.0)	
To bradycardia	0 (0)	0 (0)	
Line access time	3.3 ± 7.5	5.6 ± 11.9	0.374
Usage of inotropic	17 (44.74)	16 (57.14)	0.319

*IQR* Inter Quatile Range, *CPR* Cardiopulmonary resuscitation, *OHCA* Out-hospital cardiac arrest, *CT* Computer tomography, *MR* Magnetic Resonance, *VT* Ventricular tachycardia, *Vf* Ventricular fibrillation, *PEA* Pulseless electrical activity.

The median number of administered epinephrine in surviving patients was 2 (IQR = 1–5), while in deceased patients, it was 16.5 (IQR = 7–22) (*p* value <.001). The mean value of total CPR duration performed on surviving patients was 16.5 ± 15.8 minutes, and on deceased patients, it was 57.8 ± 36.6 minutes (*p* value < .001). Mean in-hospital CPR duration for OHCA on surviving patients was 13.6 ± 14.2 minutes, and for deceased patients, it was 53.5 ± 37.6 minutes (*p* value < .001).

The underlying disease was classified into: no history, congenital, acquired, and combined. A significant difference between surviving and deceased patients was noted (*p* value = 0.039).

Changes in cardiac rhythm during CPR were classified into none, asystole, ventriculcar tachycardia, ventricular fibrillation, pulseless electrical activity, and bradycardia. There was no change in rhythm during CPR in 76.3% of the surviving cases but only 53.3% of the deceased cases. Only 5.3% experienced asystole in the surviving cases, whereas 30% experienced it in the deceased cases (*p* value = 0.040). These are described in Table 1.

We selected these significant *p* values as predictors and further sub-analyzed them. Odd ratios (OR) are calculated and described in Table 2. For epinephrine administration, the mortality increased by 1.23 times for each increase (95% confidence interval (CI): 1.11-1.67, *p* value < .001). In categorical variable, mortality was found to increase 9.60 times when epinephrine was administered more than 10 times compared to below 10 times (95% CI: 2.99–30.79, *p* value 0.001). The mortality rate increased 1.06 times for each minute of the CPR duration (95% CI: 1.033-1.096, *p* value < 0.001). Notably, mortality increased 10.55 times when CPR duration exceeded 30 minutes (95% CI 2.048–54.367, *p* value 0.005). In-hospital CPR duration performed on OHCA patients also showed increased mortality by 1.067 times for each minute (95% CI: 1.034–1.102, *p* value < 0.001). In patients with electrocardiogram (ECG) rhythm changes during CPR, mortality increased 8.156 times when the rhythm changed to asystole in comparison to no changes occurring in the rhythm (95% CI: 1.567–42.436 vs 1.228–30.094, *p* value 0.013). The remaining variables showed no statistical relevance to prognosticating outcomes.

**Table 2:** Relationship between variables and mortality.

Variables	OR	95% CI	*P* value
Age (Month)	0.924	0.841–1.015	0.098
Sex			
Female (vs. Male)	1.329	0.495–3.567	0.572
Underlying disease			0.119
Congenital (vs. none)	1.49	0.359–6.193	0.583
Acquired (vs. none)	5.913	0.119–293.323	0.372
Combined (vs. none)	0.412	0.096–1.769	0.233
Etiology			0.684
Pulmonary origin (vs. cardiac origin)	0.323	0.053–1.981	0.22
Trauma (vs. cardiac origin)	0.185	0.001–23.062	0.493
Hypovolemic (vs. cardiac origin)	0.185	0.001–23.062	0.493
Sudden infant death syndrome (vs. cardiac origin)	0.758	0.1–5.739	0.788
Multiple organ failure (vs. cardiac origin)	0.556	0.069–4.446	0.580
Unknown (vs. cardiac origin)	0.079	0.002–3.574	0.192
Last normal to CPR (min)	0.994	0.969–1.02	0.673
Initiate CPR site			0.789
Emergency department (vs. OHCA)	1.637	0.325–8.255	0.550
General ward (vs. OHCA)	0.982	0.222–4.353	0.981
Intensive care unit (vs. OHCA)	0.695	0.188–2.566	0.585
Operation room (vs. OHCA)	0.162	0.005–5.743	0.317
CT or MRI room (vs. OHCA)	0.377	0.004–39.063	0.681
Number of epinephrine	1.233	1.111–1.368	<0.001
Categorized number of epinephrine			
More than 10 times (vs. 0 ~ 10 times)	9.6	2.994–30.785	0.001
Total CPR duration	1.064	1.033–1.096	<0.001
Categorized total CPR duration			
6 - 10 min (vs. 0 - 5 min)	0.242	0.009–6.675	0.402
11 - 15 min (vs. 0 - 5 min)	2.556	0.286–22.871	0.401
16 - 20 min (vs. 0 - 5 min)	4.599	0.204–103.528	0.337
21 - 25 min (vs. 0 - 5 min)	2.76	0.179–42.513	0.467
26 - 30 min (vs. 0 - 5 min)	4.6	0.542–39.06	0.162
More than 31 min (vs. 0 - 5 min)	10.553	2.048–54.367	0.005
In hospital CPR duration of OHCA (min)	1.067	1.034–1.102	<0.001
During on-call duty			
No (vs. Yes)	1.818	0.641–5.157	0.261
Initial rhythm			0.827
PEA (vs. asystole)	0.793	0.273–2.308	0.671
Bradycardia (vs. asystole)	0.659	0.157–2.77	0.570
Changes of rhythm			
To Asystole (vs. none)	8.156	1.567–42.436	0.013
To PEA (vs. none)	1.812	0.233–14.119	0.570
To Bradycardia (vs. none)	1.087	0.229–5.155	0.916
Line access time	1.025	0.974–1.08	0.342
Usage of inotropic	0.607	0.227–1.625	0.321

*OR* Odd ratios, *CI* Confidence Interval, *CPR* Cardiopulmonary resuscitation, *OHCA* Out-hospital cardiac arrest, *CT* Computer tomography, *MR* Magnetic Resonance, *PEA* Pulseless electrical activity.

## Discussion

In this study, we evaluated the variable factors that may impact the survival of resuscitated children aged under 24 months. We found that mortality increased with longer CPR duration and more epinephrine administration, with a cut-off value of 30 minutes of CPR and 10 times of epinephrine administration showing a significant increase in mortality. There are several studies on adults;^8–17^ one study mentioned that in-hospital mortality for adults also increased with increasing epinephrine dosage,^8^ and another study showed no survivors within 1 year after discharge for those with more than 10 times of epinephrine administration.^9^

During the study, we observed that pediatric CPR was frequently prolonged without valid reasons, with some cases recording CPR durations of up to 136 minutes. Our study indicates that indiscriminate prolongation of CPR duration has no significant benefit in improving survival rate, and suggests that CPR should be stopped at an appropriate time, even if the patient’s guardian prefers to continue. Further, we noted that the association between rhythm changes and mortality may be confounded by the fact that CPR was stopped when changed to asystole. Additional studies on the type and frequency of rhythm changes are needed.

This study has a few limitations, including a binary survival outcome. Since early termination is rare in pediatrics, patients after long durations of CPR should have their neurological function assessed to measure its impact on their quality of life using the cerebral performance category scale score, like other studies.^18^

Additionally, out-hospital CPR duration performed in OHCA was too short to analyze the effect of early administration of epinephrine and oxygen therapy. For pediatric CPR, there have been changes in early administration of epinephrine and oxygen therapy discerning OHCA corresponding to the revised PALS for 2020.^19^ In our study, intravenous line access and airways were secured rapidly in the in-hospital cardiac arrest (IHCA) cases. However, distinguishing between OHCA and IHCA was challenging owing to the short CPR duration performed out of the hospital. By virtue of 911, most patients had access to rapid transportation which allowed fast intravenous line access and advanced airway protection.

## Conclusion

In summary, there are some factors significantly related with mortality in infants after CPR, particularly, number of epinephrine administration and CPR duration. This is similar to other investigation on adult CPR cases. Further research is required in this field to prognosticate distinguishable IHCA and OHCA outcomes in the pediatric population, and furthermore, some scoring system can be validating to predict outcome of pediatric CPR.

## Data Availability

All data supporting the findings of this study are available within the paper and its Supplementary Information.
